# Regulation of mTORC1 by growth factors, energy status, amino acids and mechanical stimuli at a glance

**DOI:** 10.1186/s12970-016-0118-y

**Published:** 2016-03-01

**Authors:** Peter Bond

**Affiliations:** PeterBond.nl, Waterhoenlaan 25, Zeist, Netherlands

**Keywords:** mTORC1, Akt, Myostatin, Muscle protein synthesis

## Abstract

The mechanistic/mammalian target of rapamycin complex 1 (mTORC1) plays a pivotal role in the regulation of skeletal muscle protein synthesis. Activation of the complex leads to phosphorylation of two important sets of substrates, namely eIF4E binding proteins and ribosomal S6 kinases. Phosphorylation of these substrates then leads to an increase in protein synthesis, mainly by enhancing translation initiation. mTORC1 activity is regulated by several inputs, such as growth factors, energy status, amino acids and mechanical stimuli. Research in this field is rapidly evolving and unraveling how these inputs regulate the complex. Therefore this review attempts to provide a brief and up-to-date narrative on the regulation of this marvelous protein complex. Additionally, some sports supplements which have been shown to regulate mTORC1 activity are discussed.

## Background

The mechanistic/mammalian target of rapamycin complex 1 (mTORC1) has emerged as a key factor in regulation of skeletal muscle protein synthesis (MPS) [1]. mTORC1 is a protein complex comprised of the three core subunits mTOR, Raptor and mLST8 [2] and is regulated by several inputs, such as growth factors, energy status, amino acids and mechanical stimuli. mTOR forms the catalytic center of the two signaling complexes mTORC1 and mTORC2 [3], of which the first is primarily involved in regulation of MPS. Activation of the complex leads to phosphorylation of its two important sets of substrates which are involved in the translation of mRNA to protein. One comprising the eukaryotic initiation factor 4E (eIF4E)-binding proteins 4E-BP1 and 2. 4E-BPs inhibit the formation of the eIF4F complex which facilitates recruitment of the small (40S) ribosomal subunit to the 5’ end of mRNA [4]. Therefore, 4E-BPs inhibit mRNA translation initiation and phosphorylation by mTORC1 relieves this inhibition. The other important set of substrates of mTORC1 comprise the ribosomal S6 kinases S6K1 and 2. Phosphorylation of the S6Ks by mTORC1 activates them and resultingly modulates functions of translation initiation factors [5]. Additionally, S6Ks are thought to promote ribosome biogenesis and thereby increasing the translational capacity of the cell [6].

This manuscript attempts to provide a brief and up-to-date narrative of some important factors which regulate mTORC1 activity at the cellular level. Additionally, some sports supplements which have been shown to regulate mTORC1 activity are discussed.

## Regulation by growth factors

**Fig. 1:**
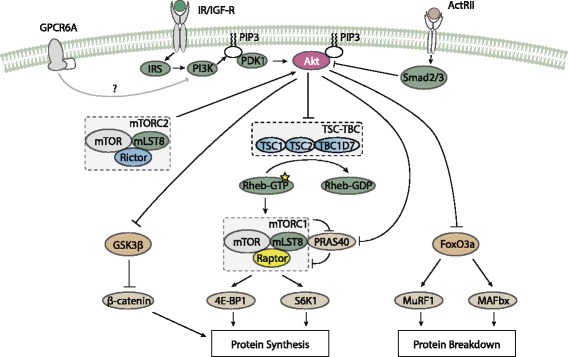
Regulation of mTORC1 by growth factors. Activation of the IR and IGF-1R leads to phosphorylation of the IRS which subsequently activate PI3K. PI3K generates PIP3 which recruits PDK1 and Akt to the plasma membrane. Akt is then activated by PDK1 and mTORC2. Activated Akt then inhibits several substrates, namely the TSC-TBC complex which functions as a negative regulator of mTORC1, GSK3 *β* which degrades *β*-catenin, FoxO3a which stimulates MuRF1 and MAFbx and PRAS40 which inhibits mTORC1. Akt activation is also induced by androgens, possibly by enhancing PI3K activity and mediated by GPCR6A. Additionally, Akt activation is inhibited by activation of ActRII receptors through activation of Smad2 and Smad3

Research examining the regulation of mTORC1 by growth factors has mainly focused on the effect of insulin and insulin-like growth factor-1. The insulin receptor (IR) and insulin-like growth factor-1 receptor (IGF-1R) both belong to the class of tyrosine kinase receptors. Activation of either receptor leads to phosphorylation of the insulin receptor substrates (IRS) proteins. This, in turn, exposes binding sites on these proteins which enable interaction with other proteins which contain a Src Homology 2 (SH2) domain. Among the SH2 domain-containing proteins is phosphatidylinositol-3-kinase (PI3K). IRS activates PI3K by associating with the SH2 domain of the kinase [7]. Activated PI3K then phosphorylates inositol phospholipids embedded in the plasma membrane on a hydroxyl group located at carbon 3. This gives rise to phosphoinositides, such as phosphatidylinositol (3,4,5)-triphosphate (PIP3). PIP3 interacts with Pleckstrin homology (PH) domain-containing proteins, thereby recruiting these to the plasma membrane. Two important PH domain-containing proteins are 3-phosphoinositide dependent protein kinase (PDK1) and Akt. The interaction of PIP3 with Akt enhances phosphorylation (and thereby activation) of the latter. Additionally, interaction of PIP3 with PDK1 leads to phosphorylation of Akt by PDK1 (Fig. 1).

Akt is considered an important upstream regulator of mTORC1 [8]. The Akt family of proteins comprises the three isoforms Akt1, Akt2 and Akt3. Akt1 and Akt2 are expressed in skeletal muscle, while Akt3 is not [9]. PDK1 phosphorylates Akt1 and Akt2 at residues Thr308 and Thr309, respectively. However, full Akt kinase activity also requires phosphorylation at a serine residue [10, 11], Ser473 and Ser473 on Akt1 and Akt2, respectively. The Rictor-containing mTOR complex mTORC2 is possibly the kinase responsible for phosphorylation of the serine residue [12]. Mechanistic studies commonly measure the phosphorylation status of Akt1 at residues Thr308 and Ser473 in order to assess Akt activity.

Myostatin, a potent negative regulator of skeletal muscle growth [13], has also been found to regulate Akt phosphorylation [14]. Myostatin is a member of the transforming growth factor- *β* superfamily and a ligand for activin type II receptors (ActRIIA and ActRIIB). After binding to its receptor, it phosphorylates and activates activin type I receptors [15]. These receptors then phosphorylate and activate the transcription factors Smad2 and Smad3 which then form a heterotrimeric complex by joining with Smad4. After formation, the complex can translocate to the nucleus where it regulates several key genes involved in skeletal muscle growth. Knockout of myostatin in animal models has been found to dramatically increase skeletal muscle fiber size and number [16–18]. In postnatal skeletal muscle, inhibition of myostatin signaling mainly affects fiber size rather than number [19, 20]. Importantly, incubation of human myoblasts with myostatin has been found to reduce Akt phosphorylation at residue Ser473 by 50 % [14]. The reduction of Akt phosphorylation by myostatin might underlie its inhibiting effect on muscle hypertrophy. Recently, researchers discovered that this effect is mediated *via* the microRNA miR-486 [21]. miR-486 increases Akt phosphorylation, likely by inhibiting phosphatase and tensin homolog (PTEN), a protein which opposes the action of PI3K by dephosphorylating PIP3 to PIP2 [22]. Myostatin negatively regulates the expression of miR-486 at the transcriptional level and therefore inhibits Akt phosphorylation mediated by PI3K.

After Akt is activated it phosphorylates several other proteins. The best researched substrates of Akt are glycogen synthase kinase 3 *β* (GSK3 *β*) [23], proline-rich Akt substrate of 40 KDa (PRAS40) [24], tuberous sclerosis complex 2 (TSC2) [25] and forkhead box class O (FoxO) proteins [26]. Both TSC2 and PRAS40 act as negative regulators of mTORC1. TSC2 forms a protein complex with TSC1 and the recently discovered protein TBC1D7 [27]. When the TSC1-TSC2-TBC1D7 (TSC-TBC) complex is formed, it inhibits mTORC1 activity by means of its GTPase-activating protein (GAP) domain [27, 28]. GTP-bound Rheb proteins (Rheb-GTP) activate mTORC1 at the lysosomal membrane [29]. The mechanism for this activation is currently unknown although interaction with the mTOR kinase domain appears to be involved [29]. By virtue of its GAP domain, the TSC-TBC complex can thus regulate the amount of Rheb-GTP and therefore mTORC1 activity. Akt phosphorylates TSC2 at multiple sites (Ser939, Ser981, Ser1130, Ser1132 and Thr1462) in order to inhibit the GAP activity of the TSC-TBC complex towards Rheb-GTP, possibly by dissociating the complex from the lysosome [30]. Moreover, it should be noted that the TSC-TBC complex has the highest affinity for Rheb-GDP rather than Rheb-GTP [30]. This might suggest a mechanism in which the complex acts to prevent the exchange of GDP for GTP in order to keep the Rheb proteins from reloading GTP. Akt further acts by relieving mTORC1 of the inhibition imposed by PRAS40. PRAS40 binds to the mTORC1 subunit Raptor, thereby inhibiting its association with substrates. PRAS40 is phosphorylated at one threonine residue (Thr246) and two serine residues (Ser181 and Ser221) [31]. The threonine residue is phosphorylated by Akt, whereas the serine residues appear to be phosphorylated by mTORC1.

GSK3 *β* is a negative regulator of the Wnt/ *β*-catenin signaling pathway as it forms a complex with other proteins and phosphorylates *β*-catenin leading to degradation of the molecule [32]. Akt phosphorylates GSK3 *β*, which inactivates the enzyme and thereby stimulates Wnt/ *β*-catenin signaling through removal of its inhibiton. *β*-catenin seems to play an important role in skeletal muscle hypertrophy by functioning as a transcription factor [33] and inhibition of GSK3 *β* stimulates hypertrophy in C2C12 myotubes [34]. The kinase has also been found to inhibit mRNA translation by blocking the GDP-GTP exhange of eIF2B [35] which is required to form a functional ternary complex for translation initiation [36].

Besides the regulation of anabolic processes through inhibition of GSK3 *β*, PRAS40 and TSC2 activity, Akt is also closely involved in inhibiting protein breakdown by modulating the activity of the FoxO family of proteins. FoxO proteins are key regulators of protein breakdown modulating ubiquitin-proteasome, as well as autophagy-lysosomal proteolytic pathways [37]. Especially the first seems important in muscle protein breakdown and two E3 ubiquitin ligases, muscle atrophy F-box (MAFbx/atrogin-1) and muscle ring finger 1 (MuRF1) [38, 39], appear to be the two main downstream effectors of FoxO signaling affecting protein breakdown. FoxO proteins are phosphorylated, and thereby inhibited, by Akt [40].

Aside from the regulation of Akt by insulin and IGF-I, some studies [41–46], but not all [47–50], suggest androgens also increase Akt phosphorylation. The large heterogeneity across these studies, such as differences in experimental animal models, differences in the type of androgen used as well as its dosage, timepoint of measurement, among others, might explain why some studies did not find an increase in Akt phosphorylation. Interestingly, one study examining the rapid effects of testosterone in cultured rat myotubes directly implicates the PI3K/Akt/mTORC1 pathway as a mediator of androgens’ effect on contractile protein synthesis [46]. Basualto-Alarcón et al., incubated the myotubes with testosterone (100 nM) and performed measurements of total Akt and phosphorylated Akt (at Ser473) 1, 5, 15, 30 and 60 m after incubation. Measurements of *α*-actin mRNA and protein were taken 6 and 12 h after incubation with testosterone and both were significantly increased, thus indicating an increase in contractile protein synthesis. Indeed, the cross-sectional area (CSA) was significantly increased after 12 h. Moreover, Akt phosphorylation was increased 15 m after incubation. When the authors inhibited PI3K, Akt or mTOR the effect on *α*-actin was blocked. As such, it appears likely that androgens exert rapid effects by activation of the PI3K/Akt/mTOR pathway. Given that PI3K operates at the cell membrane and that the effect on Akt phosphorylation occured rapidly (after 15 m), it appears highly likely that a cell membrane-localized receptor is involved. Indeed, multiple lines of evidence implicate a cell membrane-localized receptor in the rapid effects of androgens [51]. The G-protein coupled receptor (GPCR) GPRC6A has been shown to mediate a rapid signaling response, including involvement of PI3K, to testosterone [52]. In the experiment by Basualto-Alarcón et al., the addition of the androgen receptor (AR) antagonist bicalutamide blocked the increase in CSA, despite an increase in *α*-actin protein level. This indicates crosstalk between the intracellular AR and the PI3K pathway activated by testosterone. Strikingly, the intracellular AR has been shown to interact with the p85 *α* regulatory subunit of PI3K in androgen-sensitive epithelial cells, enhancing its activity [53]. However, the addition of bicalutamide to these androgen-sensitive epithelial cells blocked the androgen-induced Akt phosphorylation. This is in contrast with the experiment by Basualto-Alarcón et al., which showed that inhibition of Akt phosphorylation blocked the increase in *α*-actin protein level, whereas bicalutamide did not affect *α*-actin protein level, thus suggesting that bicalutamide did not inhibit Akt phosphorylation in this experiment. If bicalutamide also affected Akt phosphorylation in the experiment by Basualto-Alarcón et al., this should therefore be observed in *α*-actin protein level, but it remained unaltered by addition of bicalutamide. This difference between both studies might be due to the differences in cell lines and AR ligands used. Nevertheless, AR-PI3K crosstalk might, partly, underlie the absence of an increase in CSA with the addition of bicalutamide in the experiment by Basualto-Alarcón et al., despite an increase in *α*-actin. Additionally, activation of the PI3K/Akt pathway can, in turn, regulate AR activity, since Akt has been shown to post-translationally modify the AR by phosphorylation [54]. Further research might further elucidate the mechanisms through which the cell membrane-localized and intracellular AR regulate mTORC1 activity.

## Regulation by energy status

**Fig. 2:**
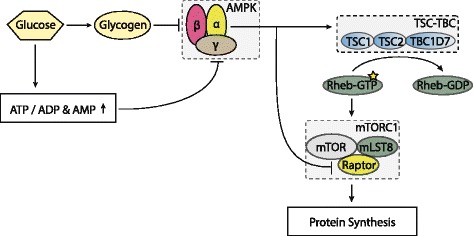
Regulation of AMPK by energy status. The *γ*-subunit interacts with the nucleotides ATP, ADP and AMP. A high ATP to ADP and AMP ratio inhibits AMPK, whereas a decrease in the ratio activates the kinase. Interaction of glycogen with the *β*-subunit allosterically inhibits AMPK activity. Activated AMPK phosphorylates TSC2 at two residues (Thr1227 and Ser1345) which are important for its activation. Moreover, activated AMPK phosphorylates Raptor at two residues (Ser722 and Ser792) which inhibits mTORC1 activity

The regulation of mTORC1 by energy status of the cell is less well described than that of growth factors and appears primarily mediated through the AMP-activated kinase (AMPK). AMPK is a heterotrimeric protein comprising a combination of *α*, *β* and *γ* subunits. Currently there are two isoforms known of both the *α* (*α*1 and *α*2) and *β* (*β*1, *β*2) subunits. There are three isoforms known of the *γ* subunit (*γ*1, *γ*2, *γ*3). The *α*-subunit functions as the catalytic subunit of the complex, whereas the other two subunits ’sense’ the energy status of the cell. The *β*-subunit can interact with glycogen [55] and the *γ*-subunit with the nucleotides adenosinetriphosphate (ATP), adenosinediphosphate (ADP) and adenosinemonophosphate (AMP) [56]. The interaction between glycogen and the *β*-subunit leads to allosteric inhibition of AMPK activity, a decrease in glycogen will therefore lead to relieve of this inhibition and thus activation of the complex. In sum, the *β* and *γ* subunits allow the kinase to measure the energy status of the cell as reflected by its glycogen content and ATP to ADP or AMP ratio. A decrease in glycogen or the ATP to ADP or AMP ratio signals a decrease in available energy to the kinase and activates it. In general, activation of AMPK promotes catabolic pathways in order to recover cellular energy homeostasis and attenuates anabolic pathways to preserve energy (Fig. 2) [56, 57].

Theoretically, the different isoforms of the subunits allows for twelve unique combinations. However, to date only three different combinations have been found in human skeletal muscle: *α*2/ *β*2/ *γ*1, *α*2/ *β*2/ *γ*3 and *α*1/ *β*2/ *γ*1 [58]. The quantitative distribution of these heterotrimeric proteins has been estimated at 15 % *α*1/ *β*2/ *γ*1, 65% *α*2/ *β*2/ *γ*1 and 20 % *α*2/ *β*2/ *γ*3. The three heterotrimers show differential regulation and effects [59]. The *α*2/ *β*2/ *γ*3 heterotrimer is rapidly activated following physical activity, whereas the other two take far longer to activate. Additionally, only the *α*1-containing heterotrimer appears to attenuate muscle growth, whereas the *α*2-containing heterotrimers do not appear to do so [60].

The antagonizing effect AMPK has on muscle growth is mediated, atleast in part, by inhibiting mTORC1 activity. AMPK phosphorylates two residues (Thr1227 and Ser1345) on TSC2 which are important for its activation [61]. TSC2 then acts to inhibit mTORC1 by formation of the TSC-TBC complex as described earlier. Moreover, Raptor, one of the proteins compromising mTORC1, has also been found to be a substrate of AMPK [62]. Phosphorylation of Raptor at residues Ser722 and Ser792 likewise inhibits mTORC1 activity.

In sum, the antagonistic effect of AMPK on mTOR is mediated through phosphorylation of TSC2 and Raptor.

## Regulation by amino acids

**Fig. 3:**
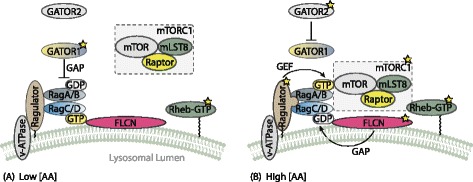
Regulation of mTORC1 by amino acids. **a** The Rags are found in their inactive state under low amino acid conditions and therefore are unable to recruit mTORC1 to the lysosomal membrane for activation by Rheb-GTP. Ragulator and v-ATPase are in their inactive state, whereas GATOR1 exerts GAP-activity towards RagA/B, ensuring an inactive state of these Rags. **b** An increase in the amino acid concentration triggers a conformational change in v-ATPase and Ragulator, which initiates GEF activity towards RagA/B of the latter. FLCN and its binding partners exhibit GAP activity towards RagC/D and thereby activating them as well. Additionally, GATOR1 its GAP activity is inhibited due to inhibition of GATOR2. These actions lead to the active heterodimer of GTP-bound RagA/B and GDP-bound RagC/D, which then recruit mTORC1 to the lysosomal surface where it can be activated by Rheb-GTP. Figure based on [99]

It should come as no surprise that the availability of the basic building blocks of protein control its synthesis. When a cell is deprived of amino acids, mTOR can be found throughout the cytoplasm, whereas addition of amino acids rapidly localizes mTOR to the peri-nuclear region of the cell, to large vesicular structures, or to both [63]. The amino acid-induced locatization is similar to that of Rab7, a late endosome-/lysosome-associated small GTPase. This suggests that amino acids might stimulate mTORC1 activity by localizing it to lysosomal surface where it can be activated by Rheb-GTP. The Ragulator-Rag complex was found responsible for targeting mTORC1 to the lysosomal surface [64]. At the lysosomal surface, mTORC1 associates with Ras-related GTPases (Rags). There are four different Rags: RagA, RagB, RagC and RagD. RagA and RagB (RagA/B) bind to RagC and RagD (RagC/D) to form heterodimeric pairs. Rags, in turn, associate with the protein complex Ragulator which is anchored in the lysosomal membrane. The interaction of Rags with mTORC1 is dependent on their guaninenucleotide binding state. In an amino acid-deprived cell, the RagA/B are bound to GDP, and the RagC/D are bound to GTP. The addition of amino acids induce a nucleotide exchange favoring the GTP bound state of RagA/B and the GDP bound state of RagC/D. The Ragulator, anchored in the lyosomal membrane, associates with Rags, therefore localizing them to the lysosomal membrane. Importantly, the Ragulator functions as a guanine nucleotide exchange factor (GEF) for RagA/B [65], thereby facilitating the exchange of GDP bound RagA/B for GTP bound RagA/B (the active form). The GEF activity of Ragulator is regulated by v-ATPase [65]. v-ATPase consumes ATP in order to pump hydrogens up their concentration gradient into the lysosome in order to maintain its acidic environment. Ragulator is associated with v-ATPase and amino acids induce a conformational change to the protein which then acts to activate Ragulator’s GEF activity. As of yet it is unclear how amino acids induce this conformational change, but the signal appears to originate from inside the lysosome due to accumulation of amino acids in its lumen (Fig. 3) [66].

Whereas Ragulator acts as a GEF for RagA/B, the GAP activity towards Rags (GATOR1) complex functions as a GAP towards RagA/B [67]. The GATOR1 complex thus exchanges the GTP for GDP of RagA/B, leading to deactivation of the Rags and subsequently inhibition of mTORC1. Another protein complex dubbed GATOR2 is responsible for inhibiting GATOR1 activity [67] and therefore relieves mTORC1 from its inhibition. The inhibiting effect of GATOR2 on GATOR1 is mediated by Sestrin proteins in response to amino acids [68]. However, it is unknown how GATOR2 mediates its inhibiting effect and how amino acids regulate the complex.

Lastly, there is evidence that the guanine nucleotide binding state of RagC/D is regulated by leucyl tRNA-synthetase (LRS), the enzyme responsible for loading tRNA with leucine. The enzyme acts as a GAP for RagD GTPase, in a leucine depedent manner [69]. However, a later study found that purified LRS did not act as a GAP for any of the Rags [70]. Instead the authors propose that folliculin tumor suppressor (FLCN) and its binding partners act as Rag-interacting proteins with GAP activity for RagC/D, leading to mTORC1 activation. Moreover, leucine specifically appears to regulate mTORC1 through Sestrin2 [71].

## Regulation by mechanical stimuli

**Fig. 4:**
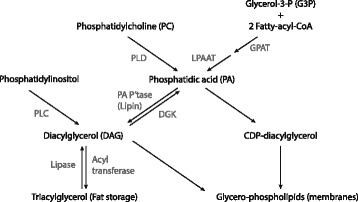
PA can be synthesized from G3P, PC and DAG. G3P is acetylated twice, requiring fatty-acyl-CoA for its acetylation. First it is acetylated by GPAT and then by LPAAT. PLD is hydrolyzed by PLD to produce PA and DAG is phosphorylated by DGK to produce PA. DAG is derived from triacylglycerols and phosphatidylinositol. PA phosphatase (PA P’tase) is responsible for dephosphorylation of PA to DAG. Various CDP-diacylglycerol synthases produce CDP-diacylglycerol from PA. Figure based on [74]

It is well known that physical activity, resistance exercise in particular, increases skeletal muscle mass in healthy persons under most conditions. Currently, two important mechanisms have been identified which regulate mTORC1 by mechanical stimuli. One of these mechanisms shows close resemblance with the PI3K/Akt-pathway in that it leads to dissociation of TSC2 from the lysosomal membrane [72]. Eccentric contractions lead to phosphorylation of TSC2 which leads to the dissociation observed. Since Rheb-GTP, the target of the TSC-TBC complex its GAP activity, is located at the lysosomal membrane, mechanical stimuli effectively prevents the GTP/GDP-exchange. Moreover, mechanical stimuli increase the levels of mTORC1 at the lysosomal membrane, further supporting its activation [72]. The mechanism for this remains uncertain (Fig. 4).

Secondly, mechanical stimuli regulate mTORC1 by regulating levels of phosphatidic acid (PA), a diacylglycerol phospholipid which has been found to directly activate mTORC1 [73]. A twofold effect mediates the stimulating effect of PA on mTORC1: i) displacing the endogeneous mTORC1 inhibitor FK506 binding protein 38 (FKBP38) through competitive inhibition, ii) allosteric activation of mTORC1.

The [PA] is regulated by five classes of enzymes [74]. Three are responsible for the synthesis of PA and two regulate its degradation. A delicate balance between the activities of these enzymes determines cellular PA levels. Glycerol-3-phosphate (G3P), phosphatidylcholine (PC) and diacylglycerol (DAG) are precursors for the biosynthesis of PA. G3P is acetylated twice in order to produce PA. First glycerol-3-phosphate acyltransferase (GPAT) catalyzes the first acetylation reaction, after which lysophosphatidic acid acyltransferase (LPAAT) catalyzes the second. PC is hydrolyzed in order to produce PA. This reaction is catalyzed by phospholipase D (PLD). For long it had been assumed PLD was crucial in mediating the mechanical stimuli-induced increase in PA. This assumption was mainly based on experiments which applied the PLD inhibitor 1-butanol, which effectively inhibited mTORC1 activity in several experiments [75]. However, later it was found that not all biological activity induced by 1-butanol could be attributed to its PLD inhibiting effect. Moreover, earlier findings already reported that PLD activity induced by mechanical stimuli poorly correlated with the cellular increase of PA [76]. Recent evidence suggests that the mechanical stimuli-induced increase of PA might be attributed to an increased synthesis from DAG rather than PC. PA is produced from DAG by phosphorylation catalyzed by diacylglycerolkinases (DGK). Many DGKs have been identified and it appears the *ζ*-isoform is primarily responsible for the mechanical stimuli-induced increase of PA [77].

The regulation of the enzymes responsible for degradation of PA are currently poorly understood.

## Sports supplements and mTORC1 signaling

In 2011, Kunkel et al. performed an elegant study to identify a compound which might help against skeletal muscle atrophy [78]. The authors screened for changes in mRNA expression in both human and rodent skeletal muscle during fasting and spinal cord injury. Both fasting and spinal cord injury involve dramatic muscle atrophy over time and this effect is driven by changes in muscle gene expression. The authors therefore hypothesized that pharmacologic compounds with opposite effects on gene expression might inhibit skeletal muscle atrophy. By querying the Connectivity Map [79] with the data they gathered, they identified ursolic acid as a potential pharmacologic compound which might inhibit skeletal muscle atrophy. After identification of the compound they continued to test its effects in mice and found it to reduce muscle atrophy and stimulate muscle hypertrophy. Interestingly, IGF-I mRNA was upregulated in skeletal muscle of the mice treated with ursolic acid. Moreover, Akt phosphorylation was also increased. The researchers also evaluated the effect of C2C12 myoblasts incubated with ursolic acid and found that, on its own, it did not increase Akt phosphorylation. However, in the presence of IGF-I ursolic acid did increase Akt phosphorylation. Similarly, ursolic acid alone did not upregulate S6K1 phosphorylation, but it did enhance IGF-I- and insulin-mediated S6K1 phosphorylation. Later research confirmed these findings and found that ursolic acid stimulates mTORC1 signaling in rat skeletal muscle [80]. This was evidenced by an increase in phosphorylation of Akt (at Thr308, but not Ser473), PRAS40 and S6K1 after resistance exercise. A recent clinical study also found an improvement in body composition and strength in sixteen Korean men with over 3 years resistance exercise experience who were supplemented ursolic acid compared to placebo [81].

Some evidence suggests that the popular ergogenic aid creatine might also stimulate mTORC1 signaling. In a double-blind placebo-controlled study, participants received either placebo or creatine for 5 days [82]. Muscle biopsies were then taken at rest, immediately after exercise, 24 and 72 h later. The phosphorylation of Akt at Ser473 and Thr308 were determined, as well as the phosphorylation of 4E-BP1 and S6K1. Surprisingly, creatine supplementation decreased Akt phosphorylation at Thr308 in rest, whereas it was unaffected immediately, 24 and 72 h post-exercise. Akt phosphorylation at Ser473 was unaffected at all time points. Similar results were obtained for 4E-BP1 and S6K1 phosphorylation: 4E-BP1 phosphorylation showed a decrease 24 h after training, while it remained unaffected at all other time points and S6K1 phosphorylation remained unchanged at all time points. Nevertheless, MHCIIA mRNA expression showed an increase immediately after exercise and MHC1 mRNA expression showed an increase during rest after creatine supplementation compared to placebo. However, another study with a similar experimental design found an increase in phosphorylated 4E-BP1 24 h after exercise in the creatine group compared to placebo, but found no difference in phosphorylated S6K1 between both groups [83]. Again, no difference was found in phosphorylated 4E-BP1 and S6K1 3 h post-exercise. Notably, an increase in IGF-I mRNA expression was also observed 24 h post-exercise in the creatine group compared to placebo. These results suggest that creatine might activate mTORC1 by increasing IGF-I activity at rest, but does not further potentiate mTORC1 signaling in the hours after exercise. Interestingly, a clinical study also found that creatine supplementation amplified the resistance exercise-induced decrease in serum myostatin [84]. Although no markers of the mTORC1 pathway were measured in this study, it might be that a decrease in serum myostatin might enhance Akt phosphorylation and thus mTORC1 activity.

The mTORC1 signaling pathway is also thought to be involved in the anabolic effects of the leucine metabolite *β*-hydroxy *β*-methylbutyrate (HMB) [85, 86]. In rats fed HMB, mTOR protein expression increased significantly compared to treatment with saline [87]. Moreover, phosphorylated S6K1 also increased significantly in the HMB treated rats compared to the control group. Similar results were obtained in an *in vitro* experiment [88]. C2C12 myoblasts were incubated with proteolysis-inducing factor (PIF, a protein which stimulates proteolysis and inhibits protein synthesis) and addition of HMB increased S6K1 phosphorylation. Notably, in the rat study no differences were found in Akt phosphorylation between both groups. However, another *in vivo* experiment did find an increase in phosphorylated Akt in differentiated C2C12 myoblasts 10 and 30 m after incubation with HMB, as well as an increase in phosphorylated mTOR 30 m after incubation [89]. These results might seem conflicting, but the measurements in the rat study were taken 15 h to 18 h after HMB supplementation. Thus it might be that the activation of Akt/mTORC1 signaling was short-lived and was therefore missed in the rat study. Interestingly, another leucine metabolite, *α*-hydroxy-isocaproic acid (HICA), has shown to increase whole lean body mass when compared to placebo in a small sample of soccer players [90]. Rats fed HICA and recovering from hindlimb immobilization also showed a sustained increase in protein synthesis and phosphorylation of S6K1 and 4E-BP1 after 14 days when compared to placebo and leucine [91]. Further research might further clarify the role of mTORC1 signaling in the anabolic effects of these leucine metabolites.

Trimethylglycine (TMG), a methyl derivate of the amino acid glycine and also known as betaine, was recently shown to improve body composition when supplemented to trained athletes [92]. TMG is hypothesized to work as an ergogenic aid by functioning as both an osmolyte as well as a methyl donor in cells [93]. In a small double-blinded crossover trial, participants underwent 2 weeks of supplementation with either TMG or placebo [94]. Before and after the 2-week period, participants performed an acute exercise session. Both before the supplementation period, as well as 10 m before and after exercise, muscle biopsies were taken from the vastus lateralis muscle. Total Akt protein content was significantly increased in the TMG group compared to placebo. There was no difference in phosphorylated Akt and S6K1 in rest, but there was a decrease in phosphorylated Akt and S6K1 after the acute exercise session in the placebo group which did not occur in the TMG group. AMPK phosphorylation at Thr172 was also measured, but there was no difference between both groups. Notably, an increase in circulating growth hormone (GH) and IGF-I concentrations was observed in the TMG group, but not in the placebo group. This makes it appealing to speculate that the increase in circulatory GH and IGF-I underlies the effect of TMG on Akt. However, it should be taken into account that local GH and IGF-I, rather than circulatory, appear to affect skeletal muscle hypertrophy [95]. Nevertheless, an *in vitro* experiment in C2C12 myoblasts showed an increase in IGF-1 receptor protein expression after incubation with TMG [96]. An increase in Akt and myosin heavy chain protein content was also observed. Taken together these observations suggest that TMG activates the IGF-I/Akt/mTORC1 pathway.

A recent study also showed that PA supplementation activated mTORC1 and improved responses in skeletal muscle hypertrophy, lean body mass, and maximal strength to resistance exercise [97]. A sample of 28 resistance trained men received either PA or placebo and took part in an 8 week periodized resistance training program. The PA group showed a larger increase in lean body mass than the placebo group and also the CSA of the rectus femoris muscle showed a larger increase in the PA group than the placebo group. The authors also performed an *in vitro* experiment assessing phosphorylation of S6K1 in C2C12 myoblasts after incubation with two different sources of PA (egg and soy). While both showed a large increase in phosphorylation of S6K1, the soy-derived PA showed the largest increase. As the authors note, the difference might be due to soy and egg derived PA having varying degrees of unsaturated and saturated fatty acid chains which influence its action. A later study carried out both an *in vivo* and *in vitro* experiment to examine the effects of PA on anabolic signaling [98]. In the *in vivo* experiment, male Wister rats received either tap water (CON), PA (PA), whey protein concentrate (WPC) or PA + WPC (PA+WPC) after an overnight fast. Samples were taken after 3 h. Ribosomal protein S6 (rpS6) phosphorylation was increased in the PA and PA+WPC groups compared to the CON group, whereas it was not increased in the WPC group. S6K1 phosphorylation was also only significantly increased compared to control in the PA+WPC group. However, while PA showed an increase in MPS compared to CON, the largest increase in MPS was observed in the WPC group. There was no synergistic effect of PA+WPC in MPS when compared to WPC alone. The authors therefore speculate that combined PA and WPC might alter mTOR pathway activation dynamics, thus shifting MPS levels to the left or right of the sampling point or that PA might interfere with WPC-induced increases in MPS. Future research might clarify this matter. Their *in vitro* experiment in C2C12 myoblasts confirmed that PA increased MPS and mTOR signaling.

## Conclusions

In the past few years our knowledge of mTORC1 regulation in skeletal muscle has increased tremendously. This review therefore attempted to provide a brief and up-to-date narrative on its regulation. Energy intake, protein intake, mechanical stumuli, as well as growth factors, have been shown to regulate the mTORC1 complex. All these elements provide signals to muscle cells which are then sensed, transduced and integrated which leads to changes in cellular functions. Ultimately, these signals are sensed by proteins such as cell surface receptors or intracellular kinases. For example, the IR senses the concentration of insulin outside the cell and relays this signal through the PI3K/Akt/mTORC1 pathway, whereas energy availability is directly relayed through AMPK by the nucleotides ATP, ADP and AMP as well as stored glycogen. Finally, these signals are integrated by the cell in order to respond accordingly by changing cellular functions such as protein synthesis and protein breakdown. mTORC1 plays a pivotal role in integrating several of these signals such as growth factors, energy status, amino acids availability and mechanical stumuli. All these signals together affect the cellular response. Sports supplements might benefit the athlete in optimizing these signals, in addition to resistance exercise training, to maximize muscle hypertrophy. While ultimately clinical trials are required to properly evaluate their effects, they are expensive and sometimes difficult to carry out. For example, it can be challenging to find enough participants which conform the criteria of interest (e.g. young adults with several years of weightlifting experience) to yield enough statistical power. Additionally, strictly controlling all variables, such as dietary intake, can be hard. This is of special concern in studies of several weeks or months of duration. Insights in the mechanistic features of sports supplements might therefore aid clinical trials by providing hypothesizes under which conditions supplements might work best, as well as which combinations of supplements might provide additive effects. Additionally, it might aid in discovering new supplements of interest. The increasing knowledge of mTORC1 regulation therefore helps to refine these matters.
